# A Unified Transcriptional, Pharmacogenomic, and Gene Dependency Approach to Decipher the Biology, Diagnostic Markers, and Therapeutic Targets Associated with Prostate Cancer Metastasis

**DOI:** 10.3390/cancers13205158

**Published:** 2021-10-14

**Authors:** Manny D. Bacolod, Francis Barany

**Affiliations:** Department of Microbiology and Immunology, Weill Cornell Medicine, New York, NY 10065, USA; barany@med.cornell.edu

**Keywords:** prostate cancer, expression, metastasis, genetic dependency, PLK1

## Abstract

**Simple Summary:**

This manuscript demonstrates how integrated bioinformatic and statistical reanalysis of publicly available genomic datasets can be utilized to identify molecular pathways and biomarkers that may be clinically relevant to metastatic prostate cancer (mPrCa) progression. The most notable observation is that the transition from primary prostate cancer to mPrCa is characterized by upregulation of processes associated with DNA replication, metastasis, and events regulated by the serine/threonine kinase PLK1. Moreover, our analysis also identified over-expressed genes that may be exploited for potential targeted therapeutics and minimally invasive diagnostics and monitoring of mPrCa. The primary data analyzed were two transcriptional datasets for tissues derived from normal prostate, primary prostate cancer, and mPrCa. Also incorporated in the analysis were the transcriptional, gene dependency, and drug response data for hundreds of cell lines, including those derived from prostate cancer tissues.

**Abstract:**

Our understanding of metastatic prostate cancer (mPrCa) has dramatically advanced during the genomics era. Nonetheless, many aspects of the disease may still be uncovered through reanalysis of public datasets. We integrated the expression datasets for 209 PrCa tissues (metastasis, primary, normal) with expression, gene dependency (GD) (from CRISPR/cas9 screen), and drug viability data for hundreds of cancer lines (including PrCa). Comparative statistical and pathways analyses and functional annotations (available inhibitors, protein localization) revealed relevant pathways and potential (and previously reported) protein markers for minimally invasive mPrCa diagnostics. The transition from localized to mPrCa involved the upregulation of DNA replication, mitosis, and PLK1-mediated events. Genes highly upregulated in mPrCa and with very high average GD (~1) are potential therapeutic targets. We showed that fostamatinib (which can target PLK1 and other over-expressed serine/threonine kinases such as AURKA, MELK, NEK2, and TTK) is more active against cancer lines with more pronounced signatures of invasion (e.g., extracellular matrix organization/degradation). Furthermore, we identified surface-bound (e.g., ADAM15, CD276, ABCC5, CD36, NRP1, SCARB1) and likely secreted proteins (e.g., APLN, ANGPT2, CTHRC1, ADAM12) that are potential mPrCa diagnostic markers. Overall, we demonstrated that comprehensive analyses of public genomics data could reveal potentially clinically relevant information regarding mPrCa.

## 1. Introduction

Prostate cancer (PrCa) is the third most common cancer in the world, with a global incidence of 1,276,106 (7.1%) and mortality of 358,989 (3.8%), according to 2018 reports [1]. Among men, PrCa is the most commonly diagnosed and deadliest in 105 and 46 countries, respectively. Mortality rates are notably higher in Sub-Saharan Africa, the Caribbean, and African Americans [2].

Our understanding of the biology, molecular pathology and genetics concerning PrCa has grown immensely over the years, particularly during the modern genomics era. According to Catalogue of Somatic Mutations in Cancer (COSMIC) (https://cancer.sanger.ac.uk/cosmic, accessed on 29 July 2021), the most commonly mutated genes in PrCa include *LRP1B* (38%), *FHIT* (23%), *TP53* (22%), *ERBB4* (22%), *CAMTA1* (20%), *ZFHX3* (17%), *GRIN2A* (16%), *ALK* (15%), *ATR* (15%), *AR* (10%), *SPOP* (9%), and *PTEN* (9%). Another common somatic aberration (~45%) is the fusion of *TMPRSS2* and *ERG*, which results in the expression of a truncated oncogenic transcription factor ERG under the control of *TMPRSS2* promoter, which is responsive to an androgen [3]. Common chromosomal aberrations include losses in 10q and 18q and gain in 8q (often in tandem with 8p-loss). These aberrations explain the frequently observed decreased expression of the tumor suppressor proteins PTEN (10q) and SMAD4 (18q) and the elevated expression of the oncoprotein MYC (8q) [4,5]. The inactivation of PTEN (either mutational or decrease in expression) leads to activation of the cancer proliferation-promoting PI3K–AKT–mTOR pathway [6]. Other tumor suppressor genes that can be repressed during PrCa progression are *APC* and *CHD1* [5]. Genome-wide comparative transcriptional analyses (primary tumors vs. normal) would also point to elevated signatures of immune cells infiltration in PrCa (e.g., increased expression of *CD28*, *CD3D*, *CTLA4*, *ICOS*) [7], which has also been reported in various pathological studies [8].

A widely used screening tool for undiagnosed PrCa is the ELISA-based PSA (prostate-specific antigen) assay. However, the diagnostic test is highly controversial given its high false-positive rate (due to high PSA levels among men with benign prostatic hyperplasia and prostatitis), the minimal benefit (~1 or fewer death per 1000 men screened, within ten years), and the adverse consequences of unnecessary treatment (such as impotence) [9,10,11].

The standard clinical management for prostate cancer includes prostatectomy (surgical removal of part or all of prostate gland), radiation therapy, and androgen deprivation therapy (ADT). ADT, intended for locally advanced cases of PrCa, is predicated on the fact that androgens (such as testosterone) can bind to and activate the androgen receptor (AR) into a transcription factor that promotes the expression of genes involved in neoplastic transformation and proliferation of prostate cells. Among the drugs used in ADT are the AR antagonists flutamide, bicalutamide, enzalutamide, darolutamide, apalutamide, and nilutamide. Nevertheless, patients treated with ADT can eventually exhibit resistance against the treatment. Various mechanisms may explain ADT resistance. These include somatic amplification or mutation in AR, as well as its androgen-independent activation [12]. AR-independent mechanisms [13] may also contribute to resistance against certain AR-inhibitors. For example, the upregulation of the wnt signaling pathway was observed in enzalutamide-resistant PrCa lines, which may explain why the focal deletion of 17q22 (which includes the gene *RNF43*, a negative regulator of Wnt pathway) is associated with enzalutamide-resistance in PrCa patient samples [14]. ADT-resistant PrCa cells may then undergo epithelial to mesenchymal transition (EMT), become locally invasive, be released as circulating tumor cells (CTCs), and overcome physical barriers to metastasize. The initial site of metastasis is often the lymph nodes next to the primary site, and it eventually extends to distant organs such as bones, lungs, and liver [5]. 

Given that the survival rate of patients with metastatic castration-resistant PrCa (mCRPC) is drastically reduced [15], it is crucial to understand the complex biology behind prostate cancer metastasis. Evidence point to bone metastasis (which is the most common) as a product of the dynamic interaction between the prostate cancer cells, the bone-forming (osteoblast), and the bone-lysing (osteoclast) cells. Prostate cancer cells may release factors influencing the balance between osteoblasts and osteoclasts activities (e.g., matrix metalloproteinases, BMP2, IGF1, PDGF, IGFBP3, VEGF, ET1, PSA, WNT, ET1, and TGFβ) toward bone formation [5,16]. Osteoblasts may then secrete factors crucial to PrCa survival and proliferation in the bone. 

We can safely assume that many aspects of PrCa metastasis are yet to be discovered and explored. This assumption leads us to design an analytical approach meant to uncover information that may eventually be relevant to diagnostics and treatment of mPrCa. The method we employed involved the integration of publicly available transcriptional, pharmacological, and genetic dependency datasets for prostate tissue (metastasis, primary tumors, normal), as well as cancer cell lines (including PrCa). The genetic dependency dataset was generated from genome-wide CRISPR (clustered regularly interspaced short palindromic repeats) knockout studies. Results described in this manuscript include the identification of potential diagnostic markers (e.g., secreted proteins for ELISA-based assays, surface protein markers that can serve as targets of radiolabeled antibodies) and therapeutic targets for metastatic PrCa, as well as prediction of molecular and signaling pathways that may drive the PrCa metastatic process. 

## 2. Materials and Methods

### 2.1. Public Genomic and Pharmacological Datasets

Several publicly available genomic and pharmacological datasets (further described in Table S1) were analyzed for this manuscript. 

#### 2.1.1. Datasets from GEO

Downloaded from Gene Expression Omnibus (GEO) (https://www.ncbi.nlm.nih.gov/geo/, accessed on 10 January 2021) are the prostate cancer Affymetrix Exon ST-generated raw CEL (intensity) files for GSE21034 [17] and GSE59745 [18] datasets. GSE21034 is an expression dataset for 131 PrCa primary tumors, 19 metastatic PrCa, and 30 normal prostates. GSE59745 was generated from 9 PrCa primary tumors, 8 metastasis, and 12 normal tissues. Using the Affymetrix Expression Console (AEC) software (now incorporated into ThermoFisher’s Transcriptome Analysis Console Software, Waltham, MA, USA) and the custom meta-probeset GPL15997 (https://www.ncbi.nlm.nih.gov/geo/query/acc.cgi?acc=GPL15997, accessed on 10 December 2020), RMA (Robust Multichip Average)-normalized expression file for 38,378 total RNA species (including protein-coding mRNAs and non-coding RNAs) was generated.

#### 2.1.2. Datasets from DepMap

Obtained from the Cancer Dependency Map (DepMap) Project portal (https://depmap.org/portal/download/, accessed on 20 February 2021) are the following datasets: (a) the RNASeq-generated Cancer Cell Line Encyclopedia (CCLE) expression (20q3 version) [19], (b) the Achilles Project’s genome-scale CRISPR knockout screen-generated gene dependency data (20q3 version) [20,21], and (c) PRISM (Profiling Relative Inhibition Simultaneously in Mixtures) primary screen drug viability (log fold change values relative to DMSO) (19q4 version) [22]. 

### 2.2. Basic Statistical Bioinformatics Tools

**Figure 1:**
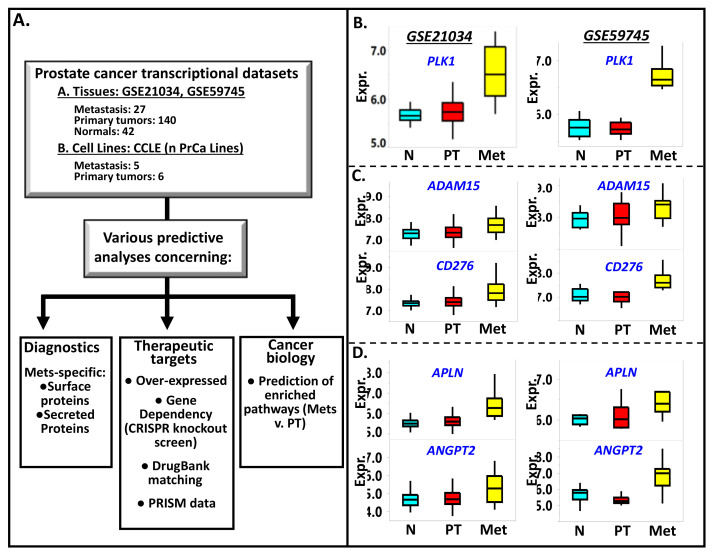
(**A**) The scheme on how integrated analyses of publicly available genomic and pharmacological data, gene/protein annotations, and pathways analytical tools are employed to identify potential diagnostic markers, therapeutic targets, and relevant biology associated with prostate cancer metastasis. Examples of genes found to be highly expressed in prostate cancer metastasis (Met) relative to primary tumors (PT) and normal prostate tissues (N) are *PLK1* (**B**), *ADAM15* and *CD276* (**C**), *APLN* and *ANGPT2* (**D**). *CD276* and *ADAM15* code for surface-bound proteins while *ANGPT2* and *APLN* code for secreted proteins. The Met v. PT *p*-values for each of these genes are (GSE21034 and GSE59745, respectively): *PLK1* (0.002, 0.002), *ADAM15* (0.002, 0.14), *CD276* (0.002, 0.004), *APLN* (0.002, 0.03), and *ANGPT2* (0.008, 0.002). On the other hand, the PT v. N expression levels of the aforementioned genes are not statistically significant, with the following *p* values (GSE21034 and GSE59745, respectively): *PLK1* (0.25, 0.71), *ADAM15* (0.09, 0.69), *CD276* (0.38, 0.92), *APLN* (0.06, 0.74), and *ANGPT2* (0.57, 0.16).

All fundamental statistical analyses (such as comparative statistics, normalization, group comparisons, biomarker discoveries, data merging, chart generations) (Figure 1A) were performed using JMP Pro 13 (Genomics) software (SAS, Cary, NC, USA) and Gene-E/Morpheus (https://software.broadinstitute.org/morpheus/, accessed on 1 June 2021) (Broad Institute, Cambridge, MA, USA).

### 2.3. Gene Annotations

Crucial to the analyses of the above datasets are the annotations of genes. Incorporated in the analyses are the following gene annotations: (a) protein subcellular locations available from Human Protein Atlas (https://www.proteinatlas.org/, accessed on 15 April 2021) [23], In-Silico Human Surfaceome (http://wlab.ethz.ch/surfaceome/, accessed on 15 April 2021) [24], the Metazoa (Human/Animal) Secretome and Subcellular Proteome Knowledge Base (MetazSecKB) (http://proteomics.ysu.edu/secretomes/animal/index.php, accessed on 20 April 2021) [25], and Gene Ontology (http://geneontology.org/, accessed on 10 April 2021) [26], (b) protein description and IDs from UniProt (https://www.uniprot.org/uniprot, accessed on 15 April 2021), and (c) drugs targeting a particular protein from DrugBank (https://go.drugbank.com/, accessed on 16 May 2021) [27]. 

### 2.4. Pathways Analyses

The prediction of associated molecular pathways was accomplished using: (a) Gene Set Enrichment Analysis (GSEA) software available through the Broad Institute website (www.broadinstitute.org/gsea/, accessed on 15 May 2021) [28]. GSEA starts with the recognition that genes associate in particular groups (or gene sets), representing pathways and functionalities, such as those defined in Biocarta (http://software.broadinstitute.org/gsea/msigdb/genesets.jsp?collection=CP:BIOCARTA/, accessed on 15 May 2021), Reactome (https://reactome.org/, accessed on 15 May 2021) [29], KEGG (https://www.genome.jp/kegg/, accessed on 15 May 2021), and Hallmark [30] and (b) Reactome over-representation analysis. A more straightforward analysis of identifying the pathways associated with a given gene was conducted via the Reactome website. The gene identifiers for a select subset of genes were entered into the Reactome analysis entry box in this analysis. The built-in program then generates a list of over-represented pathways, along with the following values for each Reactome pathway (R): (a) the number of identifiers (or genes) submitted (or found) (F) in the analysis; (b) the total (T) number of genes curated to belong to pathway R; (c) the associated probability score (*P*), calculated using Binomial Test; and (d) false discovery rate (FDR) which estimates the false positives through the Benjamini–Hochberg procedure [31].

## 3. Results

### 3.1. Prostate Cancer Metastasis Is Characterized by Upregulation of PLK1, CENPF, TOP20A, and Many Genes Involved in DNA Replication, Cell Division, and Cell Cycle

The first step in our analysis is to conduct genome-wide comparisons between the tissue subgroups (metastasis vs. primary tumors, primary tumors vs. normal prostate samples) (see Figure 1A) in the merged transcriptional dataset (GSE21034, GSE59745). The merged dataset consists of 31 metastasis, 140 primary tumors, and 41 normal prostate samples. The 31 metastatic samples have been isolated from lymph nodes (19), bone (2), brain (3), spine (3), bladder (1), colon (1), lung (1), and neck (1). Many of the genes we found to be upregulated in metastasis relative to primary tumors have previously reported roles in cancer invasiveness, metastasis, and epithelial to mesenchymal transition (EMT) (often the initial step toward invasiveness). The list includes *PLK1* (polo like kinase 1) [32] (Figure 1B), *CENPF* (centromere protein F) [33], *EXO1* (exonuclease 1) [34], *KIF20A* (kinesin family member 20A) [35], *HJURP* (Holliday junction recognition protein) [36], *PRC1*(polycomb repressor complex 1) [37], *STMN1* (stathmin 1) [38], TACC3 (transforming acidic coiled-coil containing protein 3) [39], *TPX2* (TPX2 microtubule nucleation factor) [40], *TOP2A* (DNA topoisomerase II alpha) [41], and *UBE2T* (ubiquitin-conjugating enzyme E2 T) [42]. The genes mentioned above, along with the rest of the top 300 PrCa metastasis-specific genes listed in Table S2, overlap with 14 of the 20 PrCa metastasis-upregulated genes (*CDCA8, KIF11, BUB1B, CENPE, BUB1, CDC20, TOP2A, CHEK1, CCNB2, EZH2, TPX2, CDK1, CCNA2, ALB, PLK1, AURKA, MYC, VEGFA, PTPRC, IL6*) recently identified by Gu and colleagues [43], via analysis of the transcriptional datasets GSE3325 (Affymetrix U133 plus 2.0 arrays, ThermoFisher, Waltham, MA, USA) and GSE27616 (Agilent−014850 Whole Human Genome Microarray 4 X 44K array, Agilent Technologies, Sta. Clara, CA, USA).

**Figure 2:**
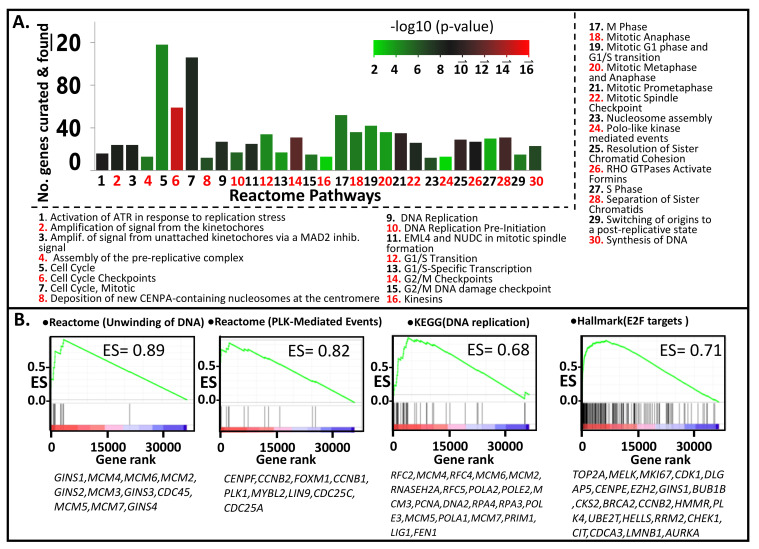
Pathways that are more enriched in metastatic relative primary tumors, identified through (**A**) Reactome analysis with the top 500 upregulated genes (metastasis vs. primary tumors), were used as input. (**B**) GSEA analysis. Shown are the GSEA plots for the pathways exhibiting the highest Enrichment Scores (ES) among the Reactome, KEGG, and Hallmark Gene Sets.

The genes *CENPF, TPX2, EXO1, HJURP*, and *TOP2A,* play pivotal roles in chromosome segregation, mitosis, and DNA replication. PLK1 is a serine/threonine kinase gene involved in mitotic regulation [32]. The top 500 genes with the highest signal-to-noise ratio (SNR; metastasis vs. PT) were subjected to Reactome over-representation analysis. Results indicated that the pathways with the lowest P score (i.e., most likely to be enriched) pertain to mitosis, cell cycle, cell cycle regulation, DNA replication, chromosomal segregation, and PLK-mediated events (Figure 2A). Similar results were obtained by applying GSEA analysis, which, unlike the Reactome over-representation analysis, the entire dataset (i.e., all the genes) was used as input. The program was run to evaluate the enrichment of molecular signatures/pathways comprising the Biocarta, Reactome, KEGG, and Hallmark databases as described in the MSigDB website (https://www.gsea-msigdb.org, accessed on 15 May 2021). The Reactome pathways which registered the highest Enrichment Scores (ES) are: “unwinding of DNA” (ES = 0.89), “polo-like kinase-mediated events” (ES = 0.82), “activation of ATR in response to replication stress” (ES = 0.79), “DNA strand elongation” (ES = 0.78), “activation of the pre-replicative complex” (ES = 0.78), “G1/S specific transcription” (ES = 0.77), “deposition of new CENPA containing nucleosomes at the centromere” (ES = 0.77), and “G0 and early G1” (ES = 0.76). The top KEGG pathways include “DNA replication” (ES = 0.68), “mismatch repair” (ES = 0.67), “homologous recombination” (ES = 0.67), “base excision repair” (ES = 0.60), and “nucleotide excision repair” (ES = 0.60). The most highly enriched Hallmark gene sets are: “E2F targets” (ES = 0.71), “G2 to M checkpoint” (ES = 0.63), “MYC targets v2” (ES = 0.56), “MYC targets v1” (ES = 0.50), “mitotic spindle” (ES = 0.50), and “DNA repair” (ES = 0.47). Shown in Figure 2B (plus more comprehensive lists in Table S3) are the enrichment plots of some of the above pathways/gene sets. The enrichment of the Reactome pathway “Unwinding of DNA” can be explained by high (and highly ranked) SNR values for several of its component genes such as *GINS1*, *MCM4*, and *MCM6*. These genes are considered the “core enrichment genes” for this gene set. The core enrichment genes for the Hallmark pathway “E2F Targets” include *TOP2A*, *MELK*, *MKI67*, *CDK1*, *DLG*, and *AURKA*.

### 3.2. PLK1-Driven Mitotic Events Are Likely Upregulated in Prostate Cancer Metastasis

**Figure 3:**
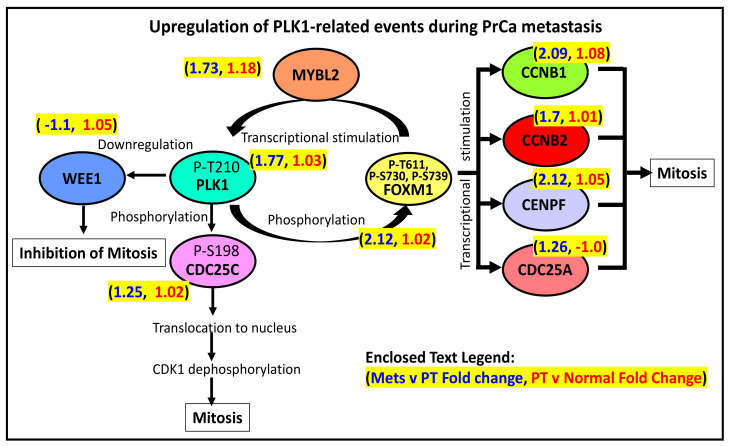
PLK1’s role in the regulation of mitosis.

As illustrated in Figure 3, the components of the Reactome pathway PLK1-related events are predominantly upregulated in PrCa metastasis relative to PT (but not in PT relative to normal prostate tissues). In this signaling pathway, a PLK1 phosphorylated at threonine 210 (or P-T210) will activate the phosphatase CDC25C, which will then translocate to the nucleus. In the nucleus, the activated CDC25C will activate the cyclin B1/CDK1 complex, which will promote early mitotic events [44]. In contrast, PLK1’s phosphorylation of the CDK1-inhibitor WEE1 may serve as the latter’s signal for degradation [45]. In addition, PLK1 (P-T210) can also phosphorylate the transcription factor FOXM1, which will then upregulate the expression of various genes involved in G2 to M transition (*CCNB1*, *CCNB2*, *CENPF*, *CDC25A*, and *PLK1*). As shown in Figure 3**,** the elevated expression of the genes mentioned above is consistent with a more active PLK1-driven signaling pathway that eventually leads to an increased mitotic rate, which is likely what happens in metastasizing prostate cancer cells [32]. One exception is *WEE1*, whose expression is lower in metastatic compared to PT tissues. It makes sense since WEE1 has a mitotic inhibitory function, as explained above.

### 3.3. Among the Metastasis-Specific Upregulated Genes Are Those Coding for Cell Surface-Bound Proteins

Positron emission tomography/computed tomography (PET/CT), which may involve the use of radiolabeled antibodies which target surface proteins, is a promising tool in diagnosing and monitoring prostate cancer metastasis and recurrence [46,47]. Hence, it is of utmost interest to identify genes for surface-bound proteins, which are also elevated in PrCa metastasis. The list of genes coding for likely surface-bound proteins was downloaded from the In Silico Human Surfaceome website (http://wlab.ethz.ch/surfaceome/, accessed on 15 August 2021). The list, consisting of 2886 unique proteins (coded by 2800 unique genes), was generated using a machine learning-based tool (SURFY) [24]. A shortlist of PrCa-metastasis upregulated genes overlapping with the Surfaceome list is shown in Table 1. The profiles of two such genes (*ADAM15* and *CD276*) are displayed in Figure 1C. *ADAM15* (ADAM metallopeptidase domain 15) codes for a protein that interacts with vascular endothelium and factors during prostate cancer metastasis [48], while the overexpression of *CD276* (or *B7H3*) proved to be a driving factor in cancer migration and invasion [49]. The other surface protein genes listed in Table 1 include: ABCC5 (ATP binding cassette subfamily C member 5) [50], *CD36* (CD36 molecule) [51], *NRP1* (neuropilin 1) [52,53], *SCARB1* (scavenger receptor class B member 1) [54], *TMEM132A* (transmembrane protein 132A) [55], *PLXNA3* (plexin A3) [56], *SERPINI1* (serpin family I member 1) [57], *ELOVL6* (ELOVL fatty acid elongase 6) [58], *LRFN1* (leucine-rich repeat and fibronectin type III domain containing 1) [59], *THY1* (Thy-1 cell surface antigen) [60], and HTR2B (5-hydroxytryptamine receptor 2B) [61]. The expression levels of *NRP1* [52,53] and *SCARB1* [54] were reported to be upregulated in metastatic mCRPCs. *PLXNA3*, a member of the plexin family of genes coding for a receptor protein, is involved in the guidance of vascular and lymphatic vessels during metastasis [56]. The rest of the genes above were also over-expressed metastatic cancers originating from breast cancer (*ABCC5*), kidney renal clear carcinoma (*LRFN1*), hepatocellular carcinoma (*ELOVL6*), and uveal melanoma (*HTR2B*).

### 3.4. Genes for Secreted Proteins Are Also Upregulated in Metastatic Prostate Cancer

The PSA test’s non-invasive and easily accessible nature is what made it a very popular early detection test for PrCa. However, its reliability is questionable because of the low specificity (high false-positive rate) resulting from the test [9,10,11]. This prompted the search for alternative ELISA-based tests to detect more reliable serum- or urine-based markers [62,63]. Recently proposed is detecting two glycoproteins (thrombospondin 1 or THBS1, and cathepsin D or CTSD) in the blood. We aimed to identify potential PrCa metastatic-specific, secreted protein markers by simply asking which of the most highly upregulated mRNAs (metastasis relative to PT) are also most likely to be translated to secretable proteins. We assume that the expressed protein is likely secreted if it passes either of the following filters: (a) the proteins are tagged as “secreted (curated)”, “secreted (highly likely)”, or “secreted (likely)”, based on information derived from MetazSecKB [25], or (b) the extracellular location is “predicted to be secreted”, according to information derived from the Human Protein Atlas [23]. As shown in Table 2, the metastatic-specific genes that may code for such proteins include *C12orf49*, *ESM1*, *APLN*, *FNDC1*, *EDA*, *ANGPT2*, *PDGFB*, and *STC2*. The expression profiles of *APLN* (apelin) and *ANGPT2* (angiopoietin 2) are illustrated in Figure 1D. Both genes have been reported as potential serum-based markers for metastatic colorectal cancer [64,65]. ANGPT2 also proved to be a serum marker of poor prognosis in lung cancer [66]. An elevated level of APLN in serum correlated with esophageal squamous cell carcinoma metastasis [67]. Other secreted protein markers, listed in Table 2, that have been demonstrated as indicators of cancer are CTHRC1 (metastatic colon cancer) [68], ESM1 (metastatic colon cancer) [69], ADAM12 (advanced stage prostate cancer) [70], PDGFB (oral squamous cell carcinoma) [71], and STC2 (laryngeal squamous cell carcinoma) [72,73].

### 3.5. Many of the Identified Metastasis-Specific Genes Have Available Inhibitors

The metastasis-upregulated genes were then matched with the DrugBank database to identify possible inhibitors [27]. Inhibitors against many of the proteins coded by these genes (Table 3) already exist. Many of the drugs have been approved by FDA for diseases other than cancer. For example, the metastasis-upregulated proteins PLK1, CDK1 (Cyclin-dependent kinase (Cdk) 1), AURKA (aurora kinase A), MELK (maternal embryonic leucine zipper kinase), and NEK2 (NIMA related kinase 2) are serine/threonine kinases that can be inhibited by fostamatinib [74,75]. This drug has been approved for the treatment of chronic immune thrombocytopenia [76]. The proteins mentioned above are also recognized as possible molecular targets in cancer. Indeed, knockdown or inhibition of PLK1 [77], CDK1 [78], AURKA [79], MELK [80,81], and NEK2 [82] resulted in reduction or repression of metastatic potential and invasiveness of various cancer types, including mCRPC and cervical cancer. The most prominent target in the list (Table 3) (as far as PrCa is concerned) is AR (androgen receptor). There are already various FDA-approved AR antagonists, including spironolactone, flutamide, bicalutamide, enzalutamide, darolutamide, apalutamide, and nilutamide. Other targets that have been associated with prostate cancer metastasis (or progression) include BIRC5/survivin [83], EZH2 [84], TOP2A [85,86], HMMR [87], LPL [88], and SSTR1 [89]. According to DrugBank, the potential inhibitors against the proteins mentioned above are reserpine and berberine for BIRC5, tazemetostat for EZH2, hyaluronic acid for HMMR, dactinomycin for TOP2A, pasireotide and somatostatin for SSTR1, and tyloxapol for LPL. Other PrCa metastasis-upregulated genes with available inhibitors, but associated with metastasis and invasiveness in other cancer types, are *ABCC5* (breast cancer) [50], *DGAT2* (gastric cancer) [90], *FEN1* (breast cancer) [91], *TYMS* (multiple cancer types including colorectal cancer) [92,93,94], *HTR2B* (uveal melanoma) [61], *RRM2* (gastric and liver cancer) [95,96], and *PNPO* (breast cancer) [97].

### 3.6. Potential Metastatic Therapeutic Targets (Such as PLK1, INCENP) Also Exhibit High Genetic Dependency

**Figure 4:**
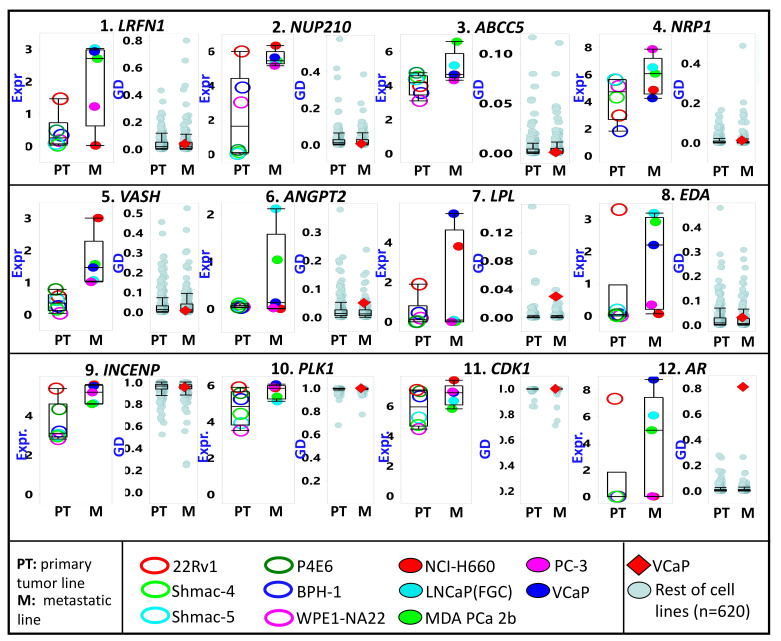
The relative cancer cell line expression (Expr) and gene dependency (GD) of some metastatic prostate cancer-upregulated genes. First row (genes 1 to 4) includes genes for surface-bound proteins. The second row (genes 5 to 8) includes genes for proteins likely secreted in serum. Cell lines are divided according to the tissue of origin (PT = primary tumor; M = metastasis). The third row (genes 9 to 12) includes genes coding for proteins with known molecular inhibitors. Only the prostate cancer lines (names listed in the bottom panel) are represented in the expression plots. All cell lines are included for the GD plots, but the lone prostate cancer line (VCap) is marked as a red diamond.

In Project Achilles, >800 cancer cell lines (*n* = 808, according to the 20Q4 data release) were subjected to a genome-wide CRISPR/Cas9 knockout screen [20,21]. The resulting sgRNA sequencing and cell viability data were then used to calculate the probability (P) that the knockout of a given gene (G) will affect the viability of a particular cell line (C). The P_GC_ score (also referred to as “gene dependency” or GD) for 18,119 genes ranged from 0 (i.e., gene knockout did not influence cell viability) to 1 (i.e., gene expression is very vital to cell viability). The average GD scores (separately for 368 PT and 253 metastatic cell lines) for each of the top 300 PrCa metastasis-upregulated can be found in Table S2. As exhibited in Figure 4, the average GD scores for the potential PrCa metastasis diagnostic markers (e.g., the surfaceome genes *LRFB1*, *NUP210*, *ABCC5*, and *NRP1*, as well as the secretome genes *VASH*, *ANGPT2*, *LPL*, and *EDA*) are closer to 0 than they are to 1. It is also noteworthy that the average expression levels of the genes mentioned above are higher among metastatic compared to PT-derived PrCa lines. In contrast, the average GD scores for *INCENP*, *PLK1*, and *CDK1* are close to 1. The expression levels of these genes are similarly higher in metastatic relative to PT PrCa cell lines. However, for the gene AR, a high gene dependency value (close to 1) is only evident in the PrCA line VCap, reflective of the gene’s unique role in PrCa progression.

### 3.7. A Tyrosine Kinase Inhibitor (Which Targets PLK1, AURKA, MELK) Exhibits Higher Efficacy against Cancer Cell Lines of Metastatic Origin

**Figure 5:**
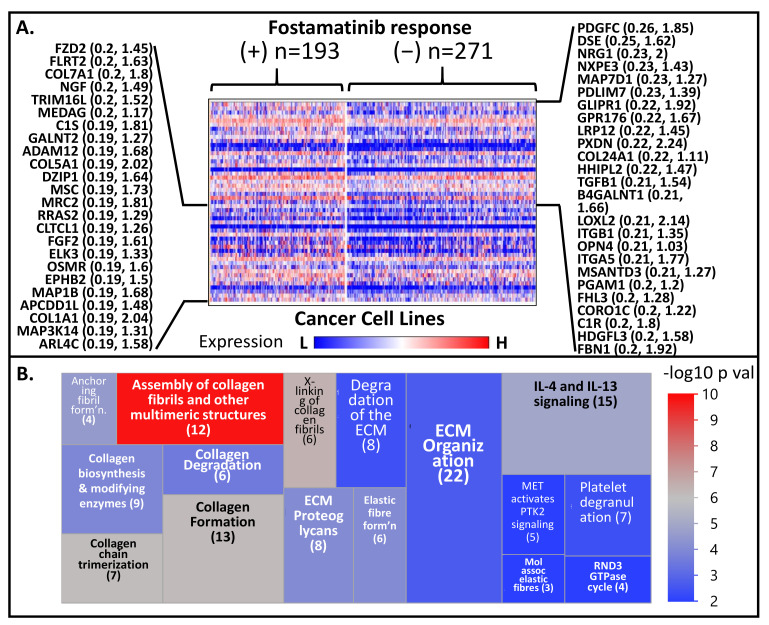
(**A**) Comparative heat map of expression of genes between relatively responsive (+) and non-responsive (–) against the serine-threonine kinase inhibitor fostamatinib. Only the genes with the highest signal-to-noise ratio (responsive vs. non-responsive) are shown. (**B**) A treemap representation of the Reactome pathways resulted when the top 150 PrCa metastasis-upregulated genes were used in the Reactome analysis. Only the top 16 pathways (the ones with the lowest Entities *p* values) are shown.

In the PRISM drug repurposing project, pools of 468 molecularly barcoded cancer cell lines were treated with 4686 drugs (majority of which have been approved for diseases other than cancer) (information taken from the 19q4 version of the public dataset) [22]. The abundance of these barcodes (relative to cells treated with DMSO) served as a measure of change in the viability of the cell lines post drug treatment. Given that there is a great deal of overlap (i.e., cell lines) between PRISM and CCLE molecular profiling datasets, it is theoretically possible to identify potential predictive or resistance markers for many of the drugs included in the PRISM project. As mentioned above, we are particularly interested in the drug fostamatinib, which targets a family of kinases including PLK1. Both genome-wide transcriptional and fostamatinib viability data are available for 464 cell lines. We arbitrarily divided the cell lines into two subgroups: (a) Group A includes cell lines that were “responsive to fostamatinib” (i.e., log fold change viability ≤ −0.5; *n* = 193), and (b) Group B covers those which were “non-responsive to fostamatinib” (i.e., log fold change between −0.5 and 0.5; *n* = 271). We then identified the highly differentiated genes between the two groups. As shown in Figure 5A (and Table S4), the upregulated genes in Group A include *COL24A1*, *COL7A1*, and many other genes related to invasion processes. Indeed, when the top 150 of such genes were subjected to Reactome analysis, we observed that the most highly dysregulated pathways (in Group A relative to Group B) are related to invasion as well as degradation of ECM, molecular pathways that are definitive signatures of metastasis (Figure 5B, Table S5). These pathways include “assembly of collagen fibrils and other multimeric structures”, “crosslinking of collagen fibrils”, “collagen formation”, “collagen chain trimerization”, “interleukin-4, and interleukin-13 signaling”, “anchoring fibril formation”, “elastic fiber formation”, “ECM proteoglycans”, “collagen biosynthesis and modifying enzymes”, “collagen degradation”, “extracellular matrix organization”, “degradation of the extracellular matrix”, “platelet degranulation”, “molecules associated with elastic fibers”, “MET activation of PTK2 signaling”, and “the RND3 GTPase cycle”. In essence, what these results suggest is that cell lines exhibiting signatures related to invasion and metastasis seem to be more responsive to inhibition of kinases such as PLK1, CDK1, MELK, and NEK.

## 4. Discussion

In recent years, the integration and reanalysis of various publicly available genomic, epigenomic, proteomic, and metabolomic datasets have been a significant source of discoveries in different areas of cancer research: from basic cancer biology to translational studies (e.g., molecular diagnostic markers, therapeutic targets). This is not surprising given the enormity of information buried in those datasets. In this report, we demonstrate the possibility of predicting the molecular pathways, non-invasive diagnostic markers, and molecular drug targets associated with the metastatic progression of prostate cancer by merely integrating various genome-wide transcriptional, gene-dependency, and pharmacological datasets.

Based on our results, it is clear that the progression from localized PrCa PT to metastasis is defined by elevated expression of many genes involved in cell division, cell cycle regulation, and DNA replication and repair process. These genes include *TPX2* (TPX2 microtubule nucleation factor), *PLK1* (polo-like kinase 1), *ANLN* (anillin actin-binding protein), EXO1 (exonuclease 1), *PRC1* (protein regulator of cytokinesis 1), *KIF20A* (kinesin family member 20A), *POC1A* (POC1 centriolar protein A), *CENPF* (centromere protein F), *HJURP* (Holliday junction recognition protein), *MCM2* and *MCM4* (minichromosome maintenance complex components 2 and 4), and *TOP2A* (DNA topoisomerase II alpha) (See Table S2). A more comprehensive pathways analysis (GSEA) or identification of common pathways/functionality signatures of genes highly upregulated in PrCa metastasis (through Reactome) would also reveal that pathways such as “unwinding of DNA”, “DNA replication”, “PLK-mediated events”, and “cell cycle checkpoints” are relatively enriched in PrCa metastasis samples. These observations are consistent with a recent report by Hsu and colleagues [98], wherein the transcription levels of *MCM* genes *2,3,4,* and *6*, which code for components of a complex involved in genome replication initiation, are elevated in Neuroendocrine PrCa (NEPC). Moreover, the inhibition of the MCM2-7 complex (by the drug ciprofloxacin) reduced cell proliferation and migration in vitro. Kauffmann and colleagues [99] reported similar observations regarding metastatic melanoma. The authors posited that a more active DNA replication and repair machinery enable metastatic melanoma to circumvent DNA damages caused by chemo- and radiation therapy.

The elevated expression of PLK1 in mPrCa may be tied to its prominent regulatory role in mitosis. A phosphorylated PLK1 can phosphorylate (and activate) the phosphatase CDC25C, the CDK1-inhibitor WEE1, and the transcription factor FOXM1. The downstream targets of these activated proteins are other proteins that regulate the G2 to M transition of cancer cells [32,44]. In addition, a recent study has shown that phosphorylation by PLK1 is also necessary to suppress the proapoptotic activity of the transcription factor FOXO1 (forkhead box protein O1) in PrCa cells [100]. Hence, targeting PLK1 by a drug can potentially inhibit or slow down PrCa’s (or any other cancer type’s) metastatic potential. This was recently demonstrated by Montaudon and colleagues in which the size of an ER-positive BrCa patient’s PT and bone metastasis-derived PDX (patient-derived xenograft) rapidly shrunk after treatment with volasertib, an inhibitor of the PLK1 (which along with AURKA and CDK1 were upregulated in the PDX) [77]. Shin and colleagues have also demonstrated that PLK1 is a target for the flavonoid genistein and that the drug was found to be selective against TP53-mutated cell lines [101]. In another recent study, fostamatinib (which inhibits PLK1 as well as other serine-threonine kinases) was shown to be effective against the prostate cancer cell line (PC3) [102]. The anti-cancer activity of fostamatinib was also evident against head and neck squamous cell carcinoma [103], hepatocellular carcinoma [104], breast cancer [105], and diffuse large B cell lymphoma [106] cell lines. Moreover, a fostamatinib derivative, NSC765691, also exhibited antiproliferative activity against the panel of NCI-60 cell lines [107]. The drug was also shown to have significant clinical activity when treating non-Hodgkin lymphoma and chronic lymphocytic leukemia patients [108]. Among the ongoing clinical trials (source: clinicaltrials.gov) which involve fostamatinib are NCT05030675 (Phase I; against lower-risk myelodysplastic syndromes or chronic myelomonocytic leukemia who have failed hypomethylating agents) and NCT03246074 (Phase I; combined with paclitaxel, against recurrent ovarian, fallopian tube, or primary peritoneal cancer).

In this current report, we also evaluated the transcriptional signatures that may be indicative of fostamatinib’s antiproliferative activity in cancer cell lines. Results indicated that fostamatinib-responsive cell lines exhibited relatively higher expression of genes belonging to the family of fibrillar and fibrillar-like collagens (*COL24A1*, *COL6A2*, *COL1A1*, *COL1A2*, *COL5A1*, and *COL6A3*). Collagens are the most abundant proteins in the ECM and provide the bulk of mechanical strength that drives cell migration [109,110,111,112]. Other genes whose expression is higher among fostamatinib-responsive cell lines are the fibrillin gene *FBN1*, the bone morphogenetic protein 1 (*BMP1*), lysyl oxidase-like 2 (*LOXL2*), the integrin genes *ITGB1* and *ITGA5*, the adamlysin gene *ADAM12*, and the growth factor genes *PDGFC* and *TGFB1*. Fibrillins are microfibrillar proteins that are also components of ECM. Integrins are heterodimer cell surface receptors utilized in downstream signaling from the ECM. The metalloprotease BMP1 cleaves the collagen precursor’s carboxy terminus, a necessary step in matrix assembly. Lysyl oxidases are enzymes needed for crosslinking collagen and elastin molecules in the ECM. Adamlysins are endopeptidases whose ability to degrade the matrix during ECM remodeling also allows cell migration during metastasis. Predictably, the results of our Reactome analysis indicated that the fostamatinib-responsive cell lines are characterized by enhanced signatures of pathways such as “assembly of collagen fibrils and other multimeric structures”, “extracellular matrix organization”, “anchoring fibril formation”, “crosslinking of collagen fibrils”, and “collagen degradation”. Overall, these observations point to the possibility that inhibitors to PLK1 (and related kinases) may help suppress prostate cancer metastasis.

Another interesting observation is the upregulation of *EZH2* (enhancer of zeste 2 polycomb repressive complex 2 subunits) in metastatic PrCa. EZH2 is the catalytic subunit of polycomb repressive complex 2 (PRC2), which silences the transcription of a given gene by the H3K27 histone. Considered a tumor suppressor, EZH2 plays a role in silencing CDH1, FOXC1, DAB2IP, and TIMP3, events linked to metastatic progression [113]. A closer look at our assembled PrCa transcriptional dataset (tissues only) indicated that *EZH2* expression negatively correlated with *TIMP3* (−0.38), *FOXC1* (R = −0.45), and *DAB2IP* (R = −0.32), but not *CDH1* (R = 0.18). Other reports indicate EZH2’s role in epigenetic silencing proapoptotic microRNAs such as miR-205 and miR-31 [84].

We were able to identify genes coding for cell surface-bound proteins, which can potentially be explored as targets for radiolabeled monoclonal antibodies for positron emission tomography (PET)-based detection of metastatic prostate cancer. These markers include *ADAM15* [48], *CD276* [49], *NRP1* [52,53], *SCARB1* [54], and *PLXNA3* [56], all of which have been reported to be overexpressed in metastatic PrCa. Elevated expression of genes such as *ABCC5* [50], *LRFN1* [59], *ELOVL6* [58], and *HTR2B* [61] have been associated with metastasis in other cancer types. Recently, PET-based detection and monitoring of metastasis cancer has utilized the following antibodies: ^111^In-labeled anti-CDH17 (gastric cancer) [114], ^177^Lu-labeled anti-CD55 (lung cancer) [115], and radio-labeled anti-ERBB2 (various labeling, including ^89^Zr, ^64^Cu, ^111^In) (breast cancer) [116]. The gene *FOLH1* (folate hydrolase 1) is of particular interest since it codes for the transmembrane metalloenzyme PSMA (prostate-specific membrane antigen). PSMA is the target for an FDA-approved ^68^Ga-based peptidomimetic radiotracer for PET imaging of PrCa [117]. Although *FOLH1* is not included in Table 1 or Table S2, the gene’s transcriptional upregulation is significant for both PrCa primary tumors (fold change and SNR relative to normal prostate are 1.42 and 0.20, respectively), and PrCa metastasis (fold change and SNR relative to primary tumors are 1.89 and 0.30, respectively).

The popular but very controversial PSA test is an ELISA-based test for the presence of PSA protein (coded by the gene *KLK3*) in serum and is intended for early detection of PrCa. Tests to detect the presence of proteins THBS1 (thrombospondin 1) and CTSD (cathepsin D) are among those being proposed as alternatives to the PSA test [63]. A non-invasive detection or monitoring of metastasis by interrogating specific proteins in patient serum (or urine) may also be feasible and backed by many publications. Several PrCa metastasis-upregulated proteins predicted to be part of the secretome have been proved experimentally as potential markers for ELISA assays. These include the proteins APLN (apelin) [64,67], ANGPT2 (angiopoietin 2) [66], CTHRC1 (collagen triple helix repeat containing 1) [68], ESM1(endothelial cell-specific molecule 1) [69], ADAM12 (ADAM metallopeptidase domain 12) [70], PDGFB (platelet-derived growth factor subunit B) [71], and STC2 (stanniocalcin 2) [72,73]. It will not be surprising if more proteins listed in Table 2 may also prove good candidates for serum-or even urine-based tests for PrCa metastasis detection and monitoring. Nonetheless, it should be pointed out that more studies are needed to ascertain the clinical utilities of these secreted proteins as diagnostic markers for mPrCa.

Apart from PLK1 (and the related serine/threonine kinases), our analysis identified a relatively long list of proteins whose inhibition can potentially (or, in theory) repress PrCa metastatic potential. It is encouraging to know that inhibitors already exist for many of these proteins, some of them FDA-approved for diseases other than cancer. Recent reports have demonstrated that inhibition of some of these proteins can potentially hinder metastasis. For example, the inhibition of the protein INCENP (by the drug reversine) led to a reduction of migration potential of colon cancer cells [118] and cell motility and invasion potential of breast cancer cells [119]. Another potential target is SSTR1(somatostatin receptor 1). The drug pasireotide, which targets this protein, exhibited efficacy against mCRPC [120] and metastatic carcinoid disease [121]. Targeting the protein BIRC5 (by the drug berberine) reduced the metastatic ability of PrCa cells [122].

The genome-wide CRISPR-generated gene dependency (GD) data incorporated in our analyses provided vital information on how a given gene’s inactivation affects cancer cells’ survival. As indicated in Table S1, *PLK1* and its related kinases (*AURKA*, *CDK1*, *MELK*, *NEK2*) have GD values very close to 1 across all cell lines (irrespective of the type of cancer or whether it is PT or metastatic origin), signifying that the protein products are essential to the survival of cancer cells, thus ideal therapeutic targets. Other genes that exhibited high GD values include *INCENP*, *TPX2*, *PRC1*, *TOP2A*, *MCM2*, and *MCM4*. The genes mentioned above are part of cells’ DNA replication and cell division machinery. An example of a PrCa-upregulated gene with a very low (close to zero) GD value is *MKI67* (a marker of proliferation Ki-67), which codes for a nuclear protein that has become a well-studied immunohistochemical marker of cancer proliferation [123]. The low GD value for MKI67 indicates that it is not an ideal therapeutic target despite being a proven marker of proliferation. Indeed, there is experimental evidence proving that altering MKI67 does not significantly affect proliferation [124]. Oher genes that surprisingly have low GD values are *SSTR1*, *ABCC5*, *PLXNA3*, *EZH2*, and *LRFN1*.

## 5. Conclusions

Although a bioinformatic exercise, this report stemmed from meticulous analyses of publicly available genomic and pharmacological data from >200 tissues and >1000 cell lines. Overall, we both validated previously reported observations and presented new and interesting observations regarding the biology, diagnostics, and molecular targeting of metastatic prostate cancer. These bioinformatic observations may also serve as a springboard for a wide array of experimental validations.

## Figures and Tables

**Table 1:** List of the most highly upregulated (metastasis vs. PT) genes coding for surface bound proteins. SNR = signal-to-noise ratio (metastasis vs. PT); permutation *p* value for all genes = 0.002; Surfaceome CSPA (Cell Surface Protein Atlas) category: 1 = high confidence, 2 = putative, 3 = unspecific.

Gene ID	Gene Description	UniProt ID	Surfaceome Category	SNR
*APLNR*	apelin receptor	P35414	1	0.95
*LRFN1*	leucine rich repeat and fibronectin type III domain containing 1	Q9P244	1	0.83
*C12orf49*	chromosome 12 open reading frame 49	Q9H741	1	0.83
*BRI3BP*	BRI3 binding protein	Q8WY22	2	0.77
*ABCA2*	ATP binding cassette subfamily A member 2	Q9BZC7	2	0.77
*NUP210*	nucleoporin 210	Q8TEM1	1	0.76
*RHBDF2*	rhomboid 5 homolog 2	Q6PJF5	1	0.73
*TMEM63C*	transmembrane protein 63C	Q9P1W3	2	0.71
*DNASE1L1*	deoxyribonuclease 1 like 1	P49184	3	0.68
*RTN4R*	reticulon 4 receptor	Q9BZR6	1	0.68
*ELOVL6*	ELOVL fatty acid elongase 6	Q9H5J4	2	0.68
*P2RY11*	purinergic receptor P2Y11	Q96G91	1	0.67
*MGAT4B*	alpha-1,3-mannosyl-glycoprotein 4-beta-N-acetylglucosaminyltransferase B	Q9UQ53	3	0.67
*TMEM132A*	transmembrane protein 132A	Q24JP5	1	0.67
*TOR3A*	torsin family 3 member A	Q9H497	3	0.66
*ABCC5*	ATP binding cassette subfamily C member 5	O15440	2	0.65
*HTR2B*	5-hydroxytryptamine receptor 2B	P41595	1	0.64
*SCARB1*	scavenger receptor class B member 1	Q8WTV0	1	0.64
*PLXNA3*	plexin A3	P51805	1	0.64
*TTYH2*	tweety family member 2	Q9BSA4	1	0.63
*SLC46A1*	solute carrier family 46 member 1	Q96NT5	1	0.63
*NRP1*	neuropilin 1	O14786	1	0.62
*ADAM12*	ADAM metallopeptidase domain 12	O43184	1	0.62
*ADAM15*	ADAM metallopeptidase domain 15	Q13444	1	0.59
*TENM4*	teneurin transmembrane protein 4	Q6N022	2	0.58
*TMEM104*	transmembrane protein 104	Q8NE00	2	0.57
*TMEM94*	transmembrane protein 94	Q12767	2	0.56
*SERPINI1*	serpin family I member 1	Q99574	1	0.56
*COL1A1*	collagen type I alpha 1 chain	P02452	1	0.55
*SLC29A1*	solute carrier family 29 member 1 (Augustine blood group)	Q99808	1	0.55
*THY1*	Thy-1 cell surface antigen	P04216	1	0.54
*PLOD3*	procollagen-lysine,2-oxoglutarate 5-dioxygenase 3	O60568	3	0.53
*AGRN*	agrin	O00468	1	0.53
*CD276*	CD276 molecule	Q5ZPR3	1	0.52
*MPHOSPH9*	M-phase phosphoprotein 9	Q99550	3	0.52
*CD36*	CD36 molecule	P16671	2	0.51

**Table 2:** List of the most highly upregulated (metastasis vs. PT) genes coding for secreted proteins. SNR = signal-to-noise ratio (Metastasis vs. PT); permutation *p* value for all genes = 0.002.

Gene ID	Gene Description	UniProt ID	Secretion Status	Location (HPA)	SNR
*C12orf49*	chromosome 12 open reading frame 49	Q9H741	Secreted (curated)		0.83
*ESM1*	endothelial cell specific molecule 1	Q9NQ30	Secreted (curated)		0.81
*APLN*	apelin	Q9ULZ1	Secreted (curated)		0.80
*FNDC1*	fibronectin type III domain containing 1	Q4ZHG4	Secreted (curated)	Secreted	0.78
*EDA*	ectodysplasin A	Q92838	Secreted (curated)		0.74
*PLA2G15*	Phospholipase A2 group XV	Q8NCC3	Lysosome, Secreted (curated)		0.68
*LPL*	lipoprotein lipase	P06858	Plasma membrane, Secreted (curated)	Secreted	0.68
*C5orf55*	uncharacterized protein EXOC3-AS1	Q8N2 × 6	Secreted (curated)		0.64
*C3orf67*	chromosome 3 open reading frame 67	Q6ZVT6	Secreted (likely)		0.63
*ANGPT2*	angiopoietin 2	O15123	Secreted (curated)		0.63
*ADAM12*	ADAM metallopeptidase domain 12	O43184	Secreted (curated)	Secreted	0.61
*CTHRC1*	collagen triple helix repeat containing 1	Q96CG8	Secreted (curated)	Secreted	0.60
*MICOS13*	mitochondrial contact site and cristae organizing system subunit 13	Q5XKP0	Secreted (likely)		0.59
*ART4*	ADP-ribosyltransferase 4 (Dombrock blood group)	DOK1		Secreted	0.59
*C12orf73*	chromosome 12 open reading frame 73	Q69YU5	Secreted (curated)	Secreted	0.58
*MAP3K9*	mitogen-activated protein kinase kinase kinase 9	P80192	Secreted (likely)		0.58
*SAMD1*	sterile alpha motif domain containing 1	Q6SPF0	Cytoplasm, Secreted (curated)		0.58
*PCYOX1L*	prenylcysteine oxidase 1 like	Q8NBM8	Secreted (curated)	Secreted	0.57
*STC2*	stanniocalcin 2	O76061	Secreted (curated)	Secreted	0.57
*PDGFB*	platelet derived growth factor subunit B	P01127	Secreted (curated)	Secreted	0.56
*SERPINI1*	serpin family I member 1	Q99574	Secreted (curated)	Secreted	0.56
*VASH1*	vasohibin 1	Q7L8A9	Secreted (curated)		0.56
*IL31*	interleukin 31	Q6EBC2	Secreted (curated)		0.56

**Table 3:** List of the most highly upregulated (metastasis vs. PT) genes coding for proteins with known inhibitors according to Drug Bank. SNR = signal-to-noise ratio (metastasis vs. PT); permutation *p*-value for all genes = 0.002.

Gene ID	Gene Description	UniProt ID	SNR	Inhibitors (Partial List; *Italic* = Approved Drug)
*PLK1*	polo like kinase 1	P53350	1.1	*Fostamatinib,* 3-[3-chloro-5-(5-{[(1S)-1-phenylethyl] amino}rplisoxazolo [5,4-c]pyridin-3-yl)phenyl]propan-1-ol)
*CIT*	citron rho-interacting serine/threonine kinase	O14578	1.0	*Fostamatinib*
*TOP2A*	DNA topoisomerase II alpha	P11388	0.97	*Doxorubicin, Dactinomycin, Etoposide,* Fleroxacin
*RRM2*	ribonucleotide reductase regulatory subunit M2	P31350	0.96	*Cladribine, Gallium nitrate*
*SSTR1*	somatostatin receptor 1	P30872	0.95	*Pasireotide*, *Somatostatin*, *Lutetium Lu 177 dotatate*
*TK1*	thymidine kinase 1	P04183	0.94	Dithioerythritol, Thymidine 5′-triphosphate
*CCNA2*	cyclin A2	P20248	0.92	4-(2,4-Dimethyl-Thiazol-5-Yl)-Pyrimidin-2-Ylamine, 6-O-Cyclohexylmethyl Guanine
*MELK*	maternal embryonic leucine zipper kinase	Q14680	0.92	*Fostamatinib*
*DGAT2*	diacylglycerol O-acyltransferase 2	Q96PD7	0.88	*Omega-3-carboxylic acids*
*INCENP*	inner centromere protein	Q9NQS7	0.85	Reversine
*NEK2*	NIMA related kinase 2	P51955	0.85	*Fostamatinib,* 5-[(Z)-(5-Chloro-2-oxo-1,2-dihydro-3H-indol-3-ylidene)methyl]-N,2,4-trimethyl-1H-pyrrole-3-carboxamide
*KIF2C*	kinesin family member 2C	Q99661	0.84	Phosphoaminophosphonic Acid-Adenylate Ester
*BIRC5*	baculoviral IAP repeat containing 5	O15392	0.78	*Reserpine, Berberine*
*HMMR*	hyaluronan mediated motility receptor	O75330	0.75	*Hyaluronic acid*
*TTK*	TTK protein kinase	P33981	0.74	*Fostamatinib,* Dithioerythritol, Thymidine 5′-triphosphate
*KIF11*	kinesin family member 11	P52732	0.74	3-[(5s)-1-Acetyl-3-(2-Chlorophenyl)-4,5-Dihydro-1h-Pyrazol-5-Yl]Phenol
*TYMS*	thymidylate synthetase	P04818	0.73	*Raltitrexed,Floxuridine,Trifluridine, Gemcitabine,* Monastrol, 10-Propargyl-5,8-Dideazafolic Acid, Deoxyuridine monophosphate
*CDK1*	cyclin dependent kinase 1	P06493	0.72	*Fostamatinib,* Indirubin-3′-monoxime, Olomoucine, Hymenialdisine
*AURKA*	aurora kinase A	O14965	0.72	*Fostamatinib*, Olomoucine, Hymenialdisine
*CHEK1*	checkpoint kinase 1	O14757	0.71	*Fostamatinib,* CHIR-124, N-{5-[4-(4-methylpiperazin-1-yl)phenyl]-1H-pyrrolo [2,3-B]pyridin-3-yl}nicotinamide
*CAD*	carbamoyl-phosphate synthetase 2, aspartate transcarbamylase, and dihydroorotase	P27708	0.71	Sparfosic acid
*EZH2*	enhancer of zeste 2 polycomb repressive complex 2 subunit	Q15910	0.70	*Tazemetostat*
*CA2*	carbonic anhydrase 2	P00918	0.69	*Benzthiazide, Cyclothiazide,* p-Hydroxymercuribenzoic acid, 4-Flourobenzenesulfonamide
*FEN1*	flap structure-specific endonuclease 1	P39748	0.69	*Iron, Ferrous gluconate, Ferrous succinate, Ferrous ascorbate*
*PNPO*	pyridoxamine 5′-phosphate oxidase	Q9NVS9	0.69	*Pyridoxal phosphate, Flavin mononucleotide*, Mercaptoethanol
*RALA*	RAS like proto-oncogene A	P11233	0.68	Guanosine-5′-Diphosphate
*LPL*	lipoprotein lipase	P06858	0.68	*Tyloxapol, Omega-3-carboxylic acids, Glycyrrhizic acid*, Glycyrrhizic acid
*POLE3*	DNA polymerase epsilon 3, accessory subunit	Q9NRF9	0.67	*Cladribine*
*UNG*	uracil DNA glycosylase	P13051	0.67	4-[(1E,7E)-8-(2,6-dioxo-1,2,3,6-tetrahydropyrimidin-4-yl)-3,6-dioxa-2,7-diazaocta-1,7-dien-1-yl]benzoic acid
*CENPE*	centromere protein E	Q02224	0.66	GSK-923295
*AR*	androgen receptor	P10275	0.65	*Spironolactone, Flutamide, Bicalutamide, Enzalutamide, Darolutamide, Apalutamide, Nilutamide*
*AK8*	adenylate kinase 8	Q96MA6	0.65	Bis(Adenosine)-5′-Pentaphosphate
*RAD51*	RAD51 recombinase	Q06609	0.65	Phosphoaminophosphonic Acid-Adenylate Ester

## Data Availability

The datasets used in the study were downloaded from GEO and DepMap websites. Links to the datasets are listed in Table S1.

## Supplementary Materials

The following are available online at https://www.mdpi.com/article/10.3390/cancers13205158/s1, Table S1: List of publicly available datasets re-analyzed in the study, Table S2: Top 300 most highly upregulated genes (Mets relative to PT), Table S3: Results of GSEA Analysis (prostate cancer metastasis vs. primary tumors), Table S4: Expression levels (across all cell lines) of top 150 genes exhibiting the highest signal-to-noise ratios (“fostamatinib responsive” vs. “fostamatinib non-responsive), Table S5: The resulting top 20 Reactome pathways (the ones with the lowest Entities *p* values) when the top 150 PrCa metastasis-upregulated genes were used as input in the Reactome analysis.
