# Supplementary material for: A Unified Transcriptional, Pharmacogenomic, and Gene Dependency Approach to Decipher the Biology, Diagnostic Markers, and Therapeutic Targets Associated with Prostate Cancer Metastasis
Source: Cancers (Basel). 2021 Oct 14;13(20):5158. doi: 10.3390/cancers13205158 (PMC8534121; doi:10.3390/cancers13205158)
Supplement: Supplementary file 1 [file cancers-13-05158-s001.zip › Table S1.pdf]

Table S1. List of publicly available datasets re-analyzed in the study

**Dataset ID**	**Data Type**	**Samples Breakdown**	**Link**
**A.GSE21034**	CEL files (Affymetrix Exon ST)	●Normals (30) ●Primary Tumors (131) ●Metastasis (19)	https://www.ncbi.nlm.nih.gov/geo/query/acc.cgi?acc=GSE21034
**B.GSE59745**	CEL files (Affymetrix Exon ST)	●Normals (12) ●Primary Tumors (9) ●Metastasis (8)	https://www.ncbi.nlm.nih.gov/geo/query/acc.cgi?acc=GSE59745
**C. CCLE Expression (20q3)**	RNA Sequencing (expression, log-2)	●Primary Origin (613) ●Metastatic Origin (407)	https://depmap.org/portal/download/
**D. PRISM repurposing primary screen (19q4)**	Viability (Logfold change values relative to DMSO)	●568 cell lines, ●4686 drugs	https://depmap.org/portal/download/
**E. Achilles gene dependency (20q3)**	Genome-scale CRISPR knockout screen	●808 cell lines (368 as primary, 253 as metastasis)	https://depmap.org/portal/download/
